# Supplementary material for: The interactome of intact mitochondria by cross-linking mass spectrometry provides evidence for coexisting respiratory supercomplexes
Source: Mol Cell Proteomics. 2017 Dec 8;17(2):216–32. doi: 10.1074/mcp.RA117.000470 (PMC5795388; doi:10.1074/mcp.RA117.000470)
Supplement: Supplemental Data [file supp_RA117.000470_133922_0_supp_23978_fzffwf.zip › spectra_annotation/mito_DR_spectra_annotation/93-1-9-1-10-1.pdf]

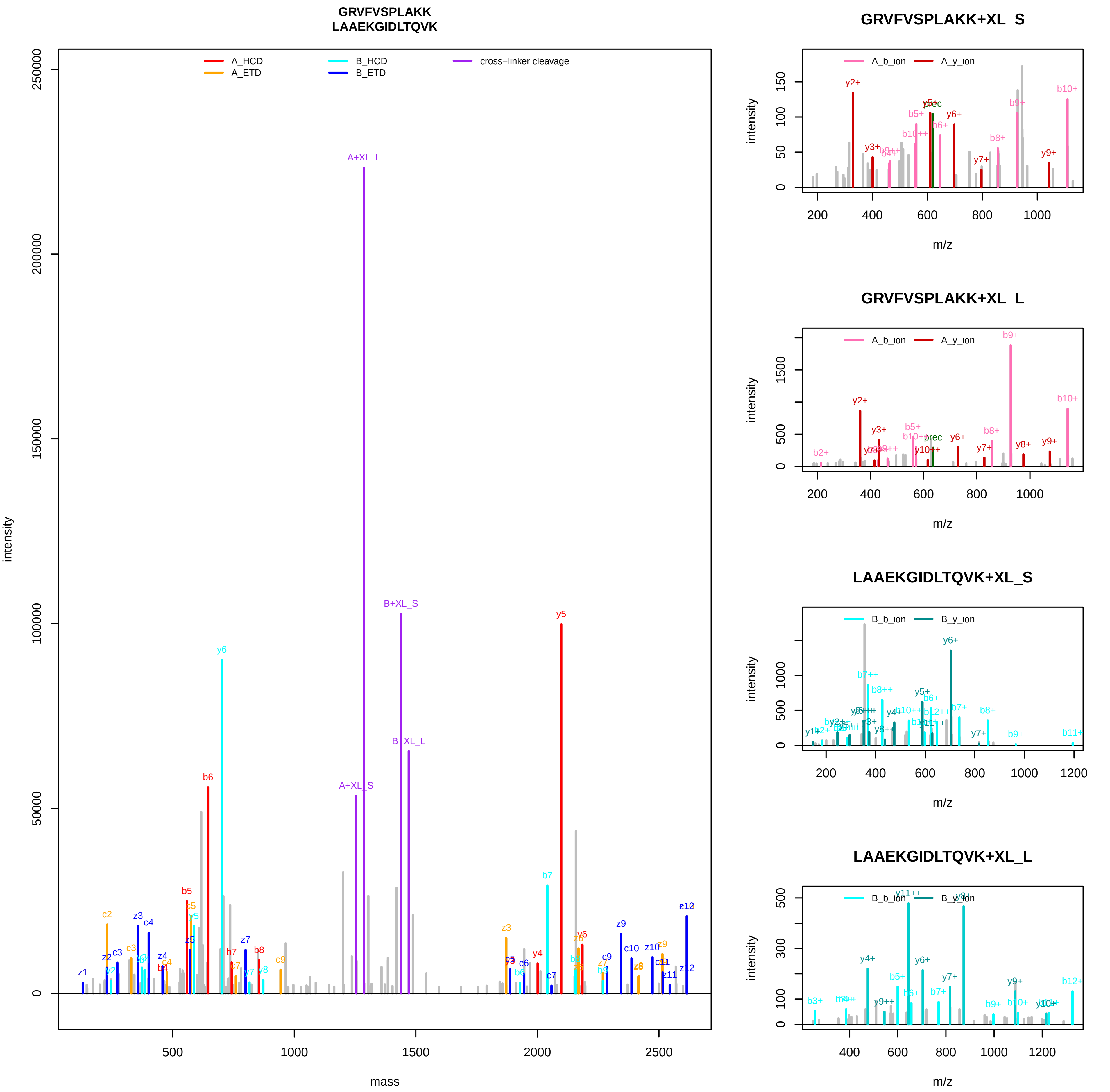

GRVFVSPLAKK
LAAEKGIDLTQVK
A_HCD
A_ETD
B_HCD
B_ETD
cross−linker cleavage
intensity
mass
0
50000
100000
150000
200000
250000
500
1000
1500
2000
2500
A+XL_L
B+XL_S
B+XL_L
A+XL_S
y5
y6
b6
b7
b5
c5
y5
z5
c2
z3
c4
z2
c3
z4
c4
b4
z7
b7
c7
b8
c9
z3
y4
y6
z6
z9
c9
c10
z10
z9
c11
z11
z12
c12
GRVFVSPLAKK+XL_S
A_b_ion
A_y_ion
y2+
y3+
y5+
prec
b5+
b6+
y6+
b10++
y7+
b8+
b9+
b10+
y9+
intensity
m/z
GRVFVSPLAKK+XL_L
A_b_ion
A_y_ion
b9+
y2+
y3+
b5+
prec
y6+
y7+
b8+
y8+
y9+
b10+
b2+
y10++
LAAEKGIDLTQVK+XL_S
B_b_ion
B_y_ion
y6+
b7++
b8++
y5+
b6+
b7+
b8+
y4+
y3+
y8++
y7+
b9+
b11+
LAAEKGIDLTQVK+XL_L
B_b_ion
B_y_ion
y11++
y8+
y4+
y6+
b5+
y7+
b7+
y9+
b12+
b3+
b4+
y9++
b9+
b10+
y10+
